# Variation of poorly ventilated lung units (silent spaces) measured by electrical impedance tomography to dynamically assess recruitment

**DOI:** 10.1186/s13054-017-1931-7

**Published:** 2018-01-31

**Authors:** Savino Spadaro, Tommaso Mauri, Stephan H. Böhm, Gaetano Scaramuzzo, Cecilia Turrini, Andreas D. Waldmann, Riccardo Ragazzi, Antonio Pesenti, Carlo Alberto Volta

**Affiliations:** 1 Department of Morphology Surgery and Experimental Medicine, Section of Anesthesia and Intensive Care, University of Ferrara, 8, Aldo Moro, 44124 Ferrara, Italy; 2 Department of Anesthesia, Critical Care and Emergency, Fondazione IRCCS (Istituto di Ricovero e Cura a Carattere Scientifico) Ca’ Granda, University of Milan, Milan, Italy; 3 Swisstom AG, Landquart, Switzerland; 4 Egalen GmbH, Lauenburg, Germany

**Keywords:** Pressure-volume curve, Electrical impedance tomography, Acute respiratory failure, Acute respiratory distress syndrome, Personalized medicine, Positive end-expiratory pressure

## Abstract

**Background:**

Assessing alveolar recruitment at different positive end-expiratory pressure (PEEP) levels is a major clinical and research interest because protective ventilation implies opening the lung without inducing overdistention. The pressure-volume (P-V) curve is a validated method of assessing recruitment but reflects global characteristics, and changes at the regional level may remain undetected. The aim of the present study was to compare, in intubated patients with acute hypoxemic respiratory failure (AHRF) and acute respiratory distress syndrome (ARDS), lung recruitment measured by P-V curve analysis, with dynamic changes in poorly ventilated units of the dorsal lung (dependent silent spaces [DSSs]) assessed by electrical impedance tomography (EIT). We hypothesized that DSSs might represent a dynamic bedside measure of recruitment.

**Methods:**

We carried out a prospective interventional study of 14 patients with AHRF and ARDS admitted to the intensive care unit undergoing mechanical ventilation. Each patient underwent an incremental/decremental PEEP trial that included five consecutive phases: PEEP 5 and 10 cmH_2_O, recruitment maneuver + PEEP 15 cmH_2_O, then PEEP 10 and 5 cmH_2_O again. We measured, at the end of each phase, recruitment from previous PEEP using the P-V curve method, and changes in DSS were continuously monitored by EIT.

**Results:**

PEEP changes induced alveolar recruitment as assessed by the P-V curve method and changes in the amount of DSS (*p* < 0.001). Recruited volume measured by the P-V curves significantly correlated with the change in DSS (*r*_s_ = 0.734, *p* < 0.001). Regional compliance of the dependent lung increased significantly with rising PEEP (median PEEP 5 cmH_2_O = 11.9 [IQR 10.4–16.7] ml/cmH_2_O, PEEP 15 cmH_2_O = 19.1 [14.2–21.3] ml/cmH_2_O; *p* < 0.001), whereas regional compliance of the nondependent lung decreased from PEEP 5 cmH_2_O to PEEP 15 cmH_2_O (PEEP 5 cmH_2_O = 25.3 [21.3–30.4] ml/cmH_2_O, PEEP 15 cmH_2_O = 20.0 [16.6–22.8] ml/cmH_2_O; *p* <0.001). By increasing the PEEP level, the center of ventilation moved toward the dependent lung, returning to the nondependent lung during the decremental PEEP steps.

**Conclusions:**

The variation of DSSs dynamically measured by EIT correlates well with lung recruitment measured using the P-V curve technique. EIT might provide useful information to titrate personalized PEEP.

**Trial registration:**

ClinicalTrials.gov, NCT02907840. Registered on 20 September 2016.

**Electronic supplementary material:**

The online version of this article (10.1186/s13054-017-1931-7) contains supplementary material, which is available to authorized users.

## Background

Ventilation of patients with acute hypoxemic respiratory failure (AHRF) and acute respiratory distress syndrome (ARDS) [1] should provide adequate gas exchange while minimizing the risk of ventilator-induced lung injury (VILI) [2, 3]. Mechanisms underlying VILI include tidal collapse and reopening of unstable lung units, overstretch of the “baby lung” [4], and heterogeneous ventilation that increases regional transpulmonary pressure [5–7]. Strategies aimed at “opening the lung and keeping it open” by means of alveolar recruitment may reduce the risk of VILI [3, 5, 8], and positive end-expiratory pressure (PEEP) set to stabilize re-aerated alveoli while avoiding overdistention might be a key aspect of protective ventilation [9]. Despite a recent large trial that showed the potential harmfulness of a maximal recruitment strategy [10], other studies in which researchers compared higher versus lower PEEP levels did not yield definitive results [11, 12]. One reason could have been the pathophysiologic heterogeneity of ARDS, with large interindividual variations in the extent and distribution of alveolar collapse that reduced clinical benefits of arbitrarily higher PEEP levels [13]. Thus, a bedside method of accurately assessing recruitment and overdistention might be fundamental to the design of future studies applying personalized “optimum” PEEP levels [2].

Lung recruitment may be evaluated by performing pressure-volume (P-V) curves of the respiratory system [14]. Several studies have validated the P-V curve method as a reliable estimate of PEEP-induced changes in aerated lung volume [15–20]. However, P-V curves are relatively complex to perform, time-consuming, noncontinuous, and not feasible in spontaneously breathing patients.

Recently, electrical impedance tomography (EIT) has been introduced as a bedside radiation-free technique that provides dynamic regional information on changes in lung aeration, ventilation, and heterogeneity [21]. EIT can identify and quantify breath-by-breath poorly ventilated lung units, also called *silent spaces*. In a recent study, researchers suggested that in postoperative patients with healthy lungs, silent spaces in the dependent lungs may indicate atelectasis, whereas increase in silent spaces in the nondependent lungs may correspond to overdistention [22].

In this study, we compared, in intubated patients with AHRF and ARDS, lung recruitment measured by P-V curve analysis with dynamic changes in poorly ventilated units of the dorsal lung (dependent silent spaces [DSSs]) assessed by EIT. We hypothesized that changes in DSS might represent a dynamic bedside measure of lung recruitment and collapse.

## Methods

### Study population

Patients with AHRF and ARDS were enrolled after we obtained approval from the Ethics Committee of the Sant’Anna Hospital, Ferrara, Italy (protocol no. 141285), and written informed consent according to local regulations. The study (registered with ClinicalTrials.gov [NCT02907840]) was conducted between December 2015 and October 2016 in accordance with the Declaration of Helsinki.

Inclusion criteria were adult patients (aged ≥18 years) who were deeply sedated and paralyzed as per clinical decision with a ratio of partial pressure of oxygen in arterial blood to fraction of inspired oxygen (PaO_2_/FiO_2_) ≤ 300 mmHg and clinical PEEP ≥ 5 cmH_2_O [1]. Exclusion criteria are listed in Additional file 1. At enrollment, we collected demographic and clinical data of each patient.

At the beginning of the study, patients were ventilated using the SERVO-i ventilator (Maquet Critical Care, Solna Sweden) in volume-controlled ventilation with tidal volume (V_T_) of 6–8 ml/kg of predicted body weight. FiO_2_ was set to obtain an arterial oxygen saturation of 90–95% [23] and was kept constant during the entire study protocol.

### EIT monitoring

EIT signals were recorded continuously throughout the study protocol using the commercially available 32-electrode Swisstom BB^2^ device (Swisstom, Landquart, Switzerland). The sampling rate used was 48 Hz. The individual’s height and weight determined the image reconstruction matrix of the respective patient [24]. EIT lung images containing 32 × 32 pixels, were displayed at the patient’s bedside. We selected four horizontal parallel regions of interest (ROIs) within the chest contour: ROI 1 (ventral), ROI 2 (central ventral), ROI 3 (central dorsal), and ROI 4 (dorsal).

### Study protocol

During the study protocol, patients were fully sedated and paralyzed using continuous infusion of propofol, morphine, and rocuronium bromide. The study protocol consisted of five consecutive phases (Additional file 1: Figure S1), each lasting 20 minutes, to reach steady-state conditions:

1. PEEP 5 cmH_2_O (PEEP5 incremental phase)
2. PEEP 10 cmH_2_O (PEEP10 incremental phase)
3. Recruitment maneuver (RM) + PEEP 15 cmH_2_O
4. PEEP 10 cmH_2_O (PEEP10 decremental phase)
5. PEEP 5 cmH_2_O (PEEP5 decremental phase)

At the end of each phase, we collected ventilation parameters, hemodynamics, and arterial blood gases and performed P-V curve analysis and change in end-expiratory lung volume (∆EELV) measurements.

### Recruitment maneuver

As previously described, an RM consisting of the application of continuous positive airway pressure of 40 cmH_2_O for 40 seconds was performed (step 3) [25, 26].

### EIT measurement

EIT data were continuously recorded and analyzed offline. The following EIT-derived parameters were measured:

1. Dependent and nondependent “silent spaces,” as previously described [22]: For each breath, pixels within the ROI showing impedance changes < 10% of the maximal impedance change were determined. The silent spaces were categorized into dependent silent spaces (DSSs) and nondependent silent spaces (NSSs) by a virtual line perpendicular to the gravity vector passing through the center of ventilation (CoV; *see details see below*). The amount of silent spaces was expressed as a percentage of the entire ROI. The final value of NSS and DSS was the average of 20 consecutive representative breaths during the last minutes of each phase.
2. CoV, which represents the geometrical focal point of the overall ventilation: This index is expressed as a percentage of the anteroposterior extension of the identified lung region, where 0% refers to ventilation occurring only in the most ventral lung region and 100% refers to ventilation in the most dorsal part [21, 27, 28] (*see also* Additional file 1).
3. Region of interest tidal volume (V_TROI_) in milliliters computed by multiplying global V_T_ by the fraction of tidal distribution of impedance signal in each ROI: Dependent and nondependent tidal volumes (V_TDEP_ and V_TNON-DEP_, respectively) were obtained by summing the V_T_ values reaching the corresponding ROIs.
4. Tidal distribution index (TDI), or anteroposterior ventilation ratio [21]: This is defined as the ratio between V_T_ delivered to the nondependent and dependent lung regions. This index is used to evaluate the homogeneity of tidal breath distribution.
5. Regional dynamic compliance for each ROI (Compl_ROIn_) and for the dependent and nondependent lung: This was calculated as follows: Compl_ROIn_ = Vt_ROIn_/driving pressure [29, 30].
6. Changes in end-expiratory lung impedance (ΔEELI) at different PEEP levels, as previously described [31].

### Lung mechanics measurements

A heated pneumotachograph (Fleisch type 2; Fleisch, Lausanne, Switzerland) was used to measure flow. V_T_ values were obtained by time integration of the flow signal. The pressure signal was recorded at the airway opening (Pao) via a rigid polyethylene catheter connected to a differential pressure transducer (200B; Raytech Instruments Inc., Vancouver, BC, Canada). Data were recorded at 100 Hz and analyzed offline to obtain the following data:

1. The P-V curve was determined by using the constant flow method [16], implying a continuous low-flow lung inflation. A third-degree polynomial equation (ΔV = a + b × Pao + c × Pao^2^) was fitted to the P-V curves.
2. ∆EELV was computed as the difference between the volume in the lung at a defined PEEP level and relaxation volume on zero PEEP, as previously described by Ranieri et al. [16, 32].
3. Recruitment/derecruitment: Recruitment was identified as the upward shift along the volume axis of the P-V curve on a given PEEP relative to the curve on the previous PEEP level and quantified as the increase in volume at the same Pao of 20 cmH_2_O [32]. Derecruitment was identified as the downward shift of the curve during decreasing PEEP levels. Recruited/derecruited volume was expressed as the difference in volume in the lungs, referring to the previous stage at the same level of Pao.
4. Respiratory system compliance (Crs), calculated as follows: Crs = V_T_/(Pplat − PEEPtot), with Pplat being plateau pressure and PEEPtot being the total positive end-expiratory pressure at the end of end-inspiratory and end-expiratory holds, respectively, each lasting 5 seconds.

### Statistical analysis

Sample size was calculated assuming correlation coefficients between changes in lung volumes assessed by EIT and spirometry with a type I error rate of 0.01 and type II error rate of 0.20 (80% power) using the sampling correlation coefficient test in MedCalc (MedCalc Software, Ostend, Belgium). The estimated correlation coefficient used in our sample size calculation was based on a previous study (*r* = 0.92) [33]. Thus, a minimum of eight patients was required. We planned to enroll an additional 30% of the estimated patients to account for patients unable to complete the entire protocol. Therefore, the total number of patients to be recruited was 14.

A nonnormal distribution was assumed, owing to the small sample size (*n* < 30). Data are presented as median and IQR. Friedman’s nonparametric test for repeated measures was used to analyze differences between the five phases. If a significant difference appeared, conditions were compared in pairwise fashion using the Wilcoxon test with the Bonferroni correction. Linear regression and Spearman’s coefficient (*r*_s_) were calculated to evaluate potential correlations between variables.

Statistical analyses were performed using IBM SPSS Statistics version 20.0 software (IBM, Armonk, NY, USA). In all statistical analyses, a two-tailed test was performed, and a *p* value ≤ 0.05 was considered statistically significant. Additional details on the methods are provided in Additional file 1.

## Results

### Patients’ characteristics

A total of 14 patients were enrolled, 12 (86%) of whom were within 1 week from diagnosis of AHRF or ARDS. The patients’ main clinical characteristics are reported in Table 1. At enrollment, all patients had a PaO_2_/FiO_2_ < 300 mmHg, with five of them (36%) having a PaO_2_/FiO_2_ < 200 mmHg. The level of clinical PEEP was 7 ± 2 cmH_2_O. Five patients (36%) showed bilateral infiltrates on chest x-rays fulfilling the Berlin ARDS criteria [1]. The protocol did not have to be interrupted in any patient because of hemodynamic instability or worsening of other clinical parameters.

**Table 1:** Patients’ main characteristics

Patient	Sex	Age (years)	BMI	SAPS II score (at ICU admission)	SOFA score (on day of study)	Etiology of acute respiratory failure	Days of intubation before study	ARDS (yes or no)	PaO_2_/FiO_2_ (mmHg)^a^	PEEP (cmH_2_O)^a^	Outcome
1	M	79	26	33	8	Thoracic trauma	7	Yes	160	8	Nonsurvivor
2	M	90	29	46	7	Sepsis	1	Yes	205	10	Survivor
3	M	71	29	30	10	Postoperative respiratory failure	2	No	230	7	Survivor
4	F	80	35	22	9	Postoperative respiratory failure	5	Yes	263	8	Survivor
5	M	69	33	30	4	Postoperative respiratory failure	2	No	168	8	Survivor
6	M	66	24	40	6	Sepsis	1	No	294	6	Survivor
7	F	85	19	38	5	Septic shock	1	No	273	7	Survivor
8	F	80	33	63	10	Sepsis in hemorrhagic shock	4	Yes	256	10	Survivor
9	F	76	24	33	8	Sepsis	2	No	258	7	Nonsurvivor
10	F	75	22	35	5	Postoperative respiratory failure	1	No	239	6	Survivor
11	F	72	26	38	10	Pneumonia	6	No	175	6	Survivor
12	F	78	35	38	11	Pneumonia	4	No	141	6	Survivor
13	M	71	34	30	9	Pancreatitis	2	Yes	98	12	Survivor
14	M	60	25	29	3	Sepsis	9	No	290	6	Survivor
Mean ± SD	7 M/7 F	75 ± 8	28 ± 5	36 ± 10	7 ± 2		4 ± 5	5 Yes/9 no	217 ± 62	7 ± 2	2 Nonsurvivors/12 survivors

*Abbreviations: BMI* Body mass index, *SAPS II* Simplified Acute Physiology Score II, *ICU* Intensive care unit, *SOFA* Sequential Organ Failure Assessment, *ARDS* Acute respiratory distress syndrome, *PaO*_*2*_*/FiO*_*2*_ Ratio of partial pressure of oxygen in arterial blood to fraction of inspired oxygen, *PEEP* Positive end-expiratory pressure

^a^Before starting the protocol (clinical)

### Effects of PEEP on respiratory mechanics, gas exchange, and hemodynamics

The effects of the different PEEP levels on respiratory mechanics, gas exchange, and hemodynamics are summarized in Table 2. Although Pplat increased with rising PEEP (*p* < 0.001), the driving pressure did not change significantly (*p* = 0.100) throughout the study protocol. Accordingly, Crs did not exhibit any significant variation (*p* = 0.100). The same was true for PaO_2_/FiO_2_ (*p* = 0.611).

**Table 2:** Lung mechanics, gas exchange, and hemodynamics data

Variable	PEEP 5 incremental phase	PEEP 10 incremental phase	PEEP 15	PEEP 10 decremental phase	PEEP 5 decremental phase	Friedman test *p* value
V_T_, ml/kg IBW	7.4 [7.0–7.8]	7.5 [7.2–7.8]	7.6 [7.4–7.8]	7.6 [7.4–7.8]	7.6 [7.4–7.8]	0.171
RR, breaths/minute	15 [14–16]	16 [15–18]	16 [14–18]	16 [14–18]	15 [14–18]	0.275
MV, L/minute	6.4 [5.8–6.6]	6.9 [6.4–7.7]	6.8 [6.5–8.0]	6.8 [6.5–8.4]	6.8 [6.4–7.8]	0.222
Ppeak, cmH_2_O	21 [19–28]	28 [26–30]^a^	34 [32–35]^a,b^	27 [25–30]^a,c^	23 [21–28]^b,c,d^	<0.001
Pplat, cmH_2_O	15 [14–19]	21 [20–23]^a^	26 [25–27]^a,b^	20 [18–22]^a,b,c^	15 [13–20]^b,c,d^	< 0.001
Driving pressure, cmH_2_O	10 [10–15]	11 [10–13]	12 [10–12]	10 [8–12]	10 [8–15]	0.100
Crs, ml/cmH_2_O	39 [32–47]	38 [34–44]	38 [34–43]	43 [35–57]	45 [30–52]	0.100
PaCO_2_, mmHg	53.8 [49.1–59.0]	53.3 [49.2–58.0]	54.0 [50.4–59.7]	52.2 [47.1–56.1]	51.8 [44.7–57.5]	0.246
PaO_2_/FiO_2_, mmHg	233 [159–286]	234 [163–279]	255 [178–292]	253 [203–320]	246 [172–304]	0.611
pH	7.33 [7.30–7.39]	7.34 [7.30–7.41]	7.32 [7.29–7.40]	7.34 [7.29–7.38]	7.34 [7.31–7.39]	0.170
MAP, mmHg	70 [66–85]	76 [67–83]	71 [64–76]	74 [66–80]	78 [70–82]	0.490
HR, beats/minute	76 [72–86]	76 [68–84]^a^	72 [67–84]^a^	73 [64–83]^a^	77 [63–85]	0.021

*Abbreviations: PEEP* Positive end-expiratory pressure, *RM* Recruitment maneuver, *V*_*T*_ Tidal volume, *IBW* Ideal body weight, *RR* Respiratory rate, *MV* Minute ventilation, *Ppeak* Peak airway pressure, *Pplat* Plateau airway pressure, *Crs* Respiratory system compliance, *PaCO*_*2*_ Partial pressure of carbon dioxide in arterial blood, *PaO*_*2*_*/FiO*_*2*_ Ratio of partial pressure of oxygen in arterial blood to fraction of inspired oxygen, *MAP* Mean arterial pressure, *HR* Heart rate

Data are expressed as median [IQR]

Wilcoxon post hoc test analysis for couples: ^a^Statistically significant difference from PEEP 5 incremental phase; ^b^Statistically significant difference from PEEP 10 incremental phase; ^c^Statistically significant difference from PEEP 15; ^d^Statistically significant difference from PEEP 10 decremental phase

### Correlation between recruitment by P-V curve and changes of silent spaces

**Fig. 1:**
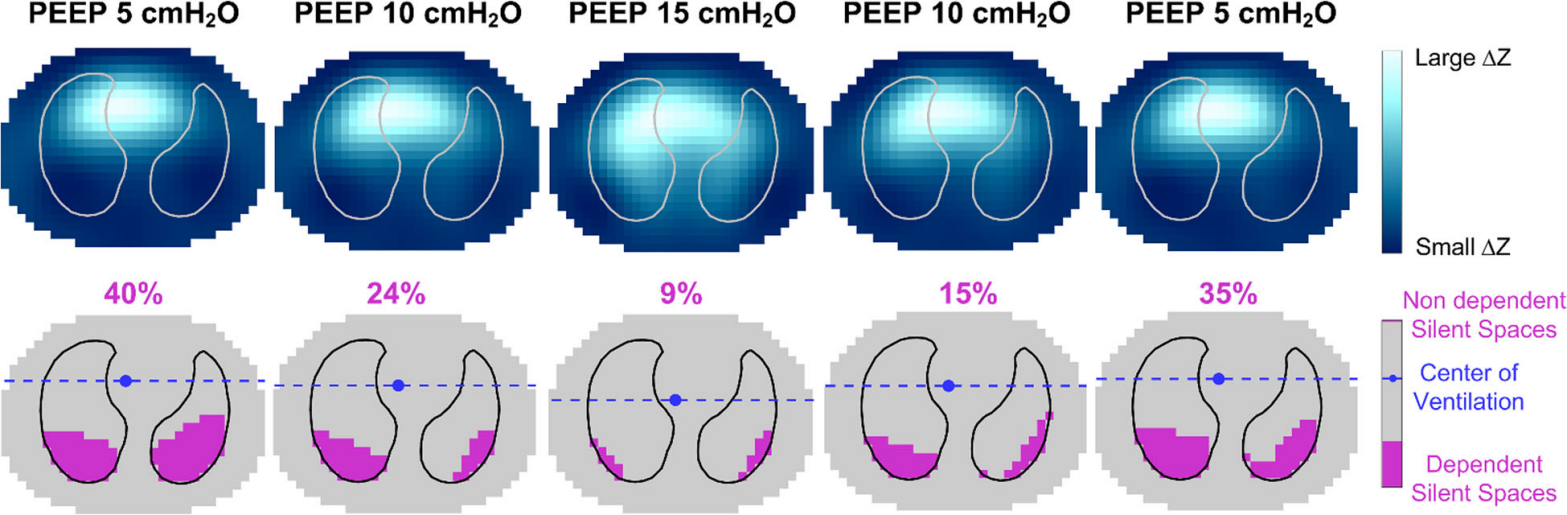
Regional impedance map and “silent spaces” values during the different study phases in a representative patient. The impedance change maps (ΔZ) during the tidal breath are shown in the upper row for each step of the protocol; in the lower row, the corresponding level of silent spaces and center of ventilation are reported. Upon incrementally increasing positive end-expiratory pressure (PEEP), the percentage of dependent silent spaces decreased, whereas the opposite was true for decreasing PEEP levels

**Fig. 2:**
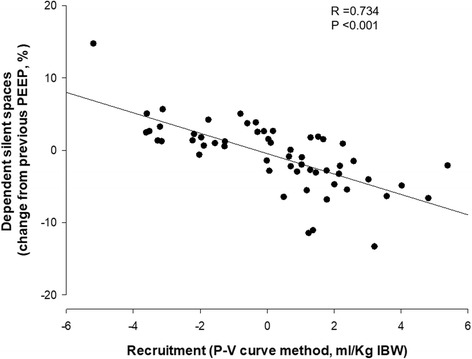
Correlation between dependent silent spaces and recruited lung volume assessed by pressure-volume (P-V) curve. The recruited volume determined by the shift in lung volumes between the P-V curves performed at different levels of positive end-expiratory pressure (PEEP) correlated inversely with the percentage change in dependent silent spaces. *IBW* Ideal body weight

PEEP had a profound effect on silent spaces: Higher levels of PEEP were associated with a decrease in silent spaces, whereas the opposite was true when reducing the level of PEEP (Table 3, Fig. 1). Changes in silent spaces occurred only in the dependent lung regions (*p* ≤ 0.001); furthermore, the RM reduced DSS compared with the same level of PEEP prior to the RM (Table 3). Incremental and decremental PEEP steps showed effects on the recruited and de-recruited lung volumes, as measured by the P-V curve method, which correlated with the EIT-derived changes of DSS (*r*_s_ = 0.734, *p* < 0.001) (Table 3, Fig. 2).

**Table 3:** Electrical impedance tomography and pressure-volume curve data

Variable	PEEP 5 incremental phase	PEEP 10 incremental phase	PEEP 15	PEEP 10 decremental phase	PEEP 5 decremental phase	Friedman test *p* value
Nondependent silent spaces, %	0.4 [0–3.3]	1.3 [0–3.9]	1.4 [0.4–4.3]	1.7 [0.5–3.0]	0.7 [0.1–2.2]	0.109
Dependent silent spaces, %	16.3 [11.7–17.9]	12.3 [9.2–15.2]^a^	8.7 [6.2–11.1]^a,b^	10.4 [8.0–12.7]^a,c^	13.6 [9.1–16.2]^a,c,d^	< 0.001
Center of ventilation ventral-dorsal, %	41.9 [38.1–48.8]	46.3 [41.8–50.8]^a^	49.4 [46.9–54.8]^a,b^	47.7 [44.3–52.3]^a,c^	43.2 [40.9–50.9]^a,c,d^	< 0.001
Regional compliance (ROI 4), ml/cmH_2_O	3.09 [1.21–4.95]	4.32 [2.92–6.03]^a^	5.90 [4.63–6.8]^a,b^	6.01 [4.53–6.53]^a,b^	4.27 [2.64–5.34]^c,d^	< 0.001
Regional compliance (ROI 3), ml/cmH_2_O	10.52 [7.74–12.27]	12.32 [8.64–14.03]^a^	13.12 [10.41–14.96]^a,b^	13.10 [10.77–17.35]^a,b^	11.25 [9.11–14.5]^a,d^	< 0.001
Regional compliance (ROI 2), ml/cmH_2_O	13.9 [12.24–18.97]	13.55 [12.82–17.06]	13.09 [11.07–15.93]^b^	14.95 [12.59–19.32]^b,c^	14.74 [11.38–20.21]	0.026
Regional compliance (ROI 1), ml/cmH_2_O	10.53 [7.51–13.27]	7.89 [5.93–9.73]	6.15 [4.63–8.15]^a,b^	7.68[6.95–10.96]^c^	10.98 [6.26–12.58]^c^	< 0.001
Tidal distribution index	1.9 [1.4–2.9]	1.3 [0.8–1.7]^a^	1.0 [0.7–1.2]^a,b^	1.3 [1.1–1.7]^a,c^	1.8 [1.4–2.2]^a,c^	< 0.001
Regional compliance dependent lung, ml/cmH_2_O	11.9 [10.4–16.7]	17.7 [12.3–19.2]^a^	19.1 [14.2–21.3]^a,b^	18.9 [15.7–22.8]^a,b^	16.0 [12.3–19.8]^a,d^	< 0.001
Regional compliance nondependent lung, ml/cmH_2_O	25.3 [21.3–30.4]	22.7 [19.5–25.8]	20.0 [16.6–22.8]^b^	24.3 [18.7–28.5]^c^	26.4 [17.5–30.7]	< 0.001
∆EELV, ml	170 [132–242]	495 [411–565]^a^	800 [638–943]^a,b^	435 [336–574]^a,c^	190 [133–262]^b,c,d^	< 0.001
∆EELI, ml	170 [105–260]	559 [404–716]^a^	1190 [903–1378]^a,b^	777 [500–930] ^a,b,c^	270 [191–410]^a,b,c,d^	< 0.001
Recruitment P-V curve, ml	Baseline	87.60 [32.20–119.00]	114.50 [71.50–171.00]	−82.20 [−164.70 to 3.00]	−101.20 [−158.50 to −28.00]	
Recruitment P-V curve, ml/kg IBW	Baseline	1.32 [0.66–2.18]	2.09 [1.05–3.22]	−1.61 [−3.26 to 0.04]	−1.65 [−2.22 to −0.59]	

*Abbreviations: PEEP* Positive end-expiratory pressure, *RM* Recruitment maneuver, *ROI* Region of interest, ∆*EELV* Change in end-expiratory lung volume, ∆*EELI* Change in end-expiratory lung impedance, *P-V* Pressure-volume, *IBW* Ideal body weight

Data are expressed as median [IQR]

Wilcoxon post-hoc analysis for couples: ^a^Statistically significant difference from PEEP 5 incremental phase; ^b^Statistically significant difference from PEEP 10 incremental phase; ^c^Statistically significant difference from PEEP 15; ^d^Statistically significant difference from PEEP 10 decremental phase

### Effects of PEEP on EIT-derived data

EIT data are summarized in Table 3. The regional distribution of ventilation changed during the protocol. By increasing the PEEP level, the CoV moved toward the dependent lung, whereas the CoV returned to the nondependent lung during the decremental PEEP steps. TDI also showed a shift of ventilation toward the dependent lung at higher PEEP levels (*p* < 0.001). Regional compliance of the dependent lung increased significantly with rising PEEP, whereas regional compliance of the nondependent lung decreased from PEEP 5 to PEEP 15 (*p* < 0.001).

ROI 4 exhibited the highest potential for recruitment because its regional compliance almost doubled. By moving from the dorsal to the ventral part of the lung, the potential for recruitment decreased toward ROI 1, in which regional compliance decreased by 60%.

## Discussion

The main findings of this study are as follows: (1) PEEP-induced changes in EIT-derived poorly ventilated areas in the dependent lungs (DSSs) correlate with recruitment determined by the P-V curve, and (2) the reduction of DSS, obtained by progressive increases of PEEP, is associated with a more homogeneous distribution of ventilation and improved regional compliance of the dependent portions of the lung; however, higher levels of PEEP were associated with lower compliance of the nondependent part of the lung, which might indicate overdistention.

In the present study, we compared the EIT imaging technique with the P-V curve of the respiratory system to explore the hypothesis that these two methods deliver similar information about PEEP-induced lung recruitment. Silent spaces are a new EIT-derived parameter aimed at identifying areas of the lung in which air content changes minimally during tidal ventilation, potentially representing collapse or overdistention [22]. We hypothesized that changes in silent spaces, representing changes in functional lung size, would also have been seen in the P-V curve as changes of recruited lung volume. Confirming our hypothesis, changes in DSS observed with EIT were statistically correlated with changes of lung volume measured by the P-V curve. Although the response to changes in PEEP and to the RM was heterogeneous in terms of lung volume and DSS changes, the two parameters changed accordingly (Fig. 2) because the increase/decrease of lung volume was associated with an opposite change of DSS (Table 3). Interestingly, the PEEP level corresponding to the lowest value of DSS was 15 cmH_2_O in 12 (86%) of the patients and 10 cmH_2_O in 2 (14%) of the patients (Additional file 1: Table S1).

The application of PEEP is a key but still challenging element of lung-protective ventilation, one of its effects being the redistribution of ventilation toward dependent lungs, which results in a more homogeneous aeration [34]. Because this technique provides information about regional lung ventilation [35], several indexes addressing ventilation homogeneity have been proposed [21], and EIT has been shown to be useful for assessing the effects of PEEP on the distribution of ventilation [36–41].

The TDI is used to evaluate the ratio of V_T_ distributed to the nondependent and dependent lung. This index is an indirect signal of the homogeneity of the tidal ventilation. Our data show that incremental PEEP levels resulted in significant recruitment and a reduction of value of TDI closer to 1, which implies more homogeneous ventilation by a shift of ventilation toward dorsal regions. This phenomenon is also demonstrated by the modification of the CoV: The increased change of the CoV toward 50% suggests that the tidal ventilation is moved toward the dependent lung regions. This index, in fact, changes significantly (*p* < 0.001) from PEEP 5 cmH_2_O (41.9%) to PEEP 15 cmH_2_O (49.4%). Interestingly, TDI indicated the most homogeneous distribution of ventilation at PEEP 15, 10, and 5 cmH_2_O in 9 (64%), 4 (28%), and 1 (7%) patients, respectively (Additional file 1: Table S1). These results show that different PEEP levels were able to achieve better lung homogeneity; indeed, tailored mechanical ventilation reducing lung inhomogeneity might decrease regional lung stress [42] and improve patient survival [43].

The use of silent spaces as a bedside method to determine the recruitment of functional lung volume has many advantages compared with the P-V curve because the monitoring is breathwise and continuous, providing local information. Furthermore, it does not require the use of high doses of sedatives or muscle relaxants.

EIT allows for assessment of regional lung mechanics on a pixel basis by tracking changes in regional lung compliance [44]. Mauri and colleagues showed that rising PEEP levels reduced lung strain and increased EELV, but at the expense of nondependent lung overinflation [39]. In our study, dependent regional compliance appeared to significantly increase rising PEEP levels, whereas nondependent regional compliance acted inversely, suggesting that higher PEEP can be associated with the risk of overdistention of that region. The determination of regional lung compliance might further support PEEP selection as the value that balances recruitment of dependent lung with “acceptable” overdistention of the nondependent one. Hence, the information obtained by EIT can be clinically relevant because the interpretation of global respiratory mechanics is often misleading in patients undergoing mechanical ventilation. In fact, global respiratory mechanics, like the static P-V curve, can only summarize overlapping information stemming from several ventilated units with different mechanical behaviors [45–47]. Indeed, the linear part of a static P-V curve may result from the overlap of already overdistended units and those that are opening during inspiration. Thus, a combination of different parameters obtained by EIT could reflect in more detail the properties of different lung regions that remain unrecognized by global assessments of respiratory mechanics.

Our study has some limitations. First, the method used to determine ΔEELV requires the removal of PEEP for a limited number of breaths; hence, we cannot exclude a potential alveolar derecruitment between steps. However, the correlation between the two methods should not have been affected, because ΔEELV was calculated at the end of data collection for each step, and its determination was always obtained during the same condition, making the influence on the quality of our data limited. Second, EIT imaging covers only the central part of the lungs (approximately 50%) close to where the EIT belt is positioned. Third, we enrolled both patients with AHRF and patients with ARDS, in keeping with previous studies [39], but this might have introduced some heterogeneity of the population. Fourth, the number of patients enrolled in this physiological study was low but was in keeping with previous studies in this field [39]. Fourth, further studies are needed to integrate silent spaces into a clinical protocol for bedside selection of personalized PEEP, despite the fact that our study does not suggest a straightforward protocol to select PEEP on the basis of DSSs. Finally, further studies are required to find out if the variation of silent spaces determined by EIT is also correlated with recruitment in spontaneously breathing patients so that these spaces could be used to set a personalized level of PEEP. However, a reference standard different from P-V curve analysis should be used in that context.

## Conclusions

Changes in EIT-derived DSSs induced by PEEP correlate with lung recruitment assessed by the P-V curve. Although the averaged maximal reduction of DSSs in our population was obtained at a PEEP of 15 cmH_2_O, worsening of regional nondependent lung compliance suggested some degree of overdistention. EIT seems to be a promising bedside tool for dynamic detection of regional changes in lung volumes due to recruitment and overdistention, potentially yielding useful information to select personalized PEEP.

## Additional file

Additional file 1: Variation of poorly ventilated lung units (silent spaces) measured by electrical impedance tomography to dynamically assess recruitment. Additional information about the manuscript methods and additional data analysis are provided. **Figure S1.** Study protocol. Study protocol consisted of five consecutive phases. **Figure S2.** Hyperinflation (%) and nondependent lung compliance (ml/cmH_2_O) during the decremental step of the protocol. Hyperinflation (%) and nondependent lung compliance (ml/cmH_2_O) during the decremental step of the protocol. The hyperinflation value is expressed as a percentage of the total pixels and is relative to the last step of the PEEP titration trial (in this case, PEEP = 5 cmH_2_O). (ZIP 178 kb)

## Abbreviations

AHRF: Acute hypoxemic respiratory failure
ARDS: Acute respiratory distress syndrome
BMI: Body mass index
Compl_ROIn_: Regional dynamic compliance for each region of interest
CoV: Center of ventilation
Crs: Respiratory system compliance
DSS: Dependent silent spaces
ΔEELI: Change in end-expiratory lung impedance
ΔEELV: Change in end-expiratory lung volume
EIT: Electrical impedance tomography
FiO_2_: Fraction of inspired oxygen
HR: Heart rate
IBW: Ideal body weight
ICU: Intensive care unit
MAP: Mean arterial pressure
MV: Minute ventilation
NSS: Nondependent silent spaces
PaCO_2_: Partial pressure of carbon dioxide in arterial blood
PPao: Pressure signal recorded at the airway opening
PaO_2_: Partial pressure of oxygen in arterial blood
PEEP: Positive end-expiratory pressure
PEEPtot: Total positive end-expiratory pressure
Pplat: Plateau pressure
P-V: Pressure-volume
RM: Recruitment maneuver
ROI: Region of interest
RR: Respiratory rate
SAPS II: Simplified Acute Physiology Score II
SOFA: Sequential Organ Failure Assessment
TDI: Tidal distribution index
VILI: Ventilator-induced lung injury
V_T_: Tidal volume
V_TDEP_: Dependent lung tidal volume
V_TNON-DEP_: Nondependent lung tidal volume
V_TROI_: Regional tidal volume
