# Supplementary material for: Variation of poorly ventilated lung units (silent spaces) measured by electrical impedance tomography to dynamically assess recruitment
Source: Crit Care. 2018 Jan 31;22:26. doi: 10.1186/s13054-017-1931-7 (PMC5793388; doi:10.1186/s13054-017-1931-7)
Supplement: Additional file 1: — Variation of poorly ventilated lung units (silent spaces) measured by electrical impedance tomography to dynamically assess recruitment. Additional information about the manuscript methods and additional data analysis are provided. Figure S1. Study protocol. Study protocol consisted of five consecutive phases. Figure S2. Hyperinflation (%) and nondependent lung compliance (ml/cmH2O) during the decremental step of the protocol. Hyperinflation (%) and nondependent lung compliance (ml/cmH2O) during the decremental step of the protocol. The hyperinflation value is expressed as a percentage of the total pixels and is relative to the last step of the PEEP titration trial (in this case, PEEP = 5 cmH2O). (ZIP 178 kb) [file 13054_2017_1931_MOESM1_ESM.zip › Supplemental_file.docx]

**Variation of poorly ventilated lung units (silent spaces) measured by electrical impedance tomography to dynamically assess recruitment**

Savino Spadaro1 MD, PhD, Tommaso Mauri2 MD, Stephan H. Böhm3 MD, Gaetano Scaramuzzo1 MD, Cecilia Turrini1 MD, Andreas D. Waldmann3 MEng, Riccardo Ragazzi1 MD, Antonio Pesenti2 MD, Carlo Alberto Volta1 MD.

**SUPPLEMENTAL DIGITAL CONTENT**

**DETAILED METHODS**

**Study population.** Patients with acute hypoxemic respiratory failure and acute respiratory distress syndrome were enrolled after obtaining approval of the ethics committee of the Sant’ Anna Hospital, Ferrara, Italy (Protocol n. 141285) written informed consent according to local regulations. The study was conducted between December 2015 and October 2016 in accordance with the Declaration of Helsinki (ClinicalTrials.gov identifier: NCT02907840).

Inclusion criteria were adult patients (age ≥18 years) deeply sedated and paralyzed as per clinical decision with a PaO2/FiO2 ≤300 mmHg and a clinical PEEP ≥5 cmH2O [E1]. Exclusion criteria were: refusal to participate, pregnancy, pulmonary cardiogenic edema, unstable hemodynamics (defined by a systolic arterial pressure of 90 mmHg or less or mean arterial pressure of 60 mmHg or less), pneumothorax, severe chronic obstructive pulmonary disease, impossibility to correctly position the EIT belt (e.g. chest drainage, surgical wound dressings), contraindications to EIT monitoring (e.g., pacemaker, automatic implantable cardioverter defibrillator).

At enrollment, we collected gender, age, body mass index (BMI), simplified acute physiology score II (SAPS II) value at ICU admission, sequential organ failure assessment (SOFA), acute respiratory failure etiology, days of mechanical ventilation, diagnosis of ARDS. In-hospital mortality was also recorded.

At the beginning of the study, patients were ventilated using a Servo-I ventilator (Maquet Critical Care, Solna Sweden) in volume controlled ventilation (VCV) with tidal volume (VT) of 6-8 ml/kg of predicted body weight (PBW). FiO2 was set to obtain an arterial oxygen saturation value of 90-95% [E2] and was kept constant during the entire study protocol.

**EIT monitoring.** Electrical impedance signals were recorded continuously throughout the study protocol using the 32-electrode commercially available device Swisstom BB2 (Swisstom, Landquart, Switzerland); the system uses a textile belt containing the electrodes placed around the chest along the 6th intercostal space. The sampling rate of the acquisition system was 48 Hz. The individual’s height and weight determined the image reconstruction matrix of each patient [E3]. EIT lung images, containing 32x32 pixels, were displayed at the patient’s bedside. We selected four horizontal parallel regions of interest (ROIs) within the chest contour: ROI1 (ventral), ROI2 (central ventral), ROI3 (central dorsal), ROI4 (dorsal).

**Study protocol.** During the study protocol, all patients were fully sedated and paralyzed using continuous infusion of propofol, morphine and rocuronium bromide.

The study protocol consisted of five consecutive phases (Figure S1), each lasting 20 minutes to reach steady state conditions:

1) PEEP 5 cmH2O (PEEP5 incremental phase)

2) PEEP 10 cmH2O (PEEP10 incremental phase)

3) Recruitment maneuver (RM) + PEEP 15 cmH2O

4) PEEP 10 cmH2O (PEEP10 decremental phase)

5) PEEP 5 cmH2O (PEEP5 decremental phase)

At the end of each phase, we collected ventilation, hemodynamics and arterial blood gas (ABG) data before performing P-V curves and delta end-expiratory lung volume (∆EELV) measurements.

**Recruitment maneuver.** As previously described, a recruitment maneuver consisting of the application of a continuous positive airway pressure of 40 cmH2O for 40 seconds was performed (step 3) [E4, E5]. This maneuver was interrupted prematurely if: 1) systolic arterial pressure (PAS) decreased below 90 mmHg or by 30 mmHg from the baseline; 2) heart rate (HR) exceeded 140 bpm or increased by 20 bpm from baseline; 3) SpO2 dropped below 90%. After recruitment maneuver, we applied a PEEP of 15 cm H2O which, according to the study by Crotti [E6], should prevent derecruitment.

**EIT measurement.** EIT data were continuously recorded and analyzed off-line on a personal computer. For calculating function-EIT images (f-EIT) of regional ventilation distribution, a GREIT-based [E7]. To calculate function EIT images (f-EIT) of regional ventilation distribution a GREIT-based reconstruction method was chosen data from twenty consecutive representative breaths were taken and reconstructed using a 3D finite element model based on an individual patients’ height, weight and gender. The following EIT-derived parameters were measured:

1. Dependent and non-dependent “silent spaces” as previously described [E8]. For each breath, pixels within the ROI showing impedance changes smaller than 10% of the maximal impedance change were determined. The silent spaces were categorized into dependent (DSS) and non-dependent (NSS) by a virtual line perpendicular to the gravity vector passing through the center of ventilation (CoV, details see below). The amount of “silent spaces” was expressed as percentage of the entire ROI. The final value of NSS and DSS was the average of 20 consecutive representative breaths during the last minutes of each phase. The difference between dependent silent spaces in two consecutive protocol steps was called ∆DSS. This variable is either positive or negative depending on whether dependent silent spaces increased or decreased while passing from one level of PEEP to another.
2. Center of ventilation (CoV) ventral-dorsal, defined as the geometrical focal point of overall ventilation as a single digit [E9, E10]expressed as a percentage of the anterior–posterior extension of the identified lung region, where 0% refers to ventilation occurring in the most ventral lung region and 100% in the most dorsal part.
3. Regional tidal volume (VTROI) in milliliters computed by multiplying global VT by the fraction of tidal distribution of impedance signal in each ROI (TDROI):

VTROIn = TDROIn ∙ VT

“n” being the ROI number. Dependent and non-dependent tidal volumes (VTDEP and VTNON-DEP respectively) were obtained by summing up the tidal volumes reaching the corresponding ROIs:

VTNON-DEP=VTROI1+VTROI2

VTDEP=VTROI3+VTROI4

1. Tidal distribution index (TDI), or anteroposterior (upper to lower) ventilation ratio [E11] defined as the ratio between tidal volume delivered to the non-dependent and to the dependent lung regions:

TDI = VTNON-DEP / VTDEP.

The distance of TDI from the unity (1-TDI) is an index of the heterogeneity of the ventilation distribution.

1. Regional dynamic compliance (ComplROI) for each ROI and for the dependent and the non-dependent lung (ComplDEP and ComplNON-DEP) was calculated as follows: ComplROIn = VtROIn / Driving Pressure [E12-E13]
2. Changes in end-expiratory lung impedance at different PEEP (ΔEELI), as previously described [E14]. We measured change in end-expiratory lung impedance between the two PEEP levels and, to obtain inflation or deflation volumes, we multiplied the impedance change by the ratio between VT expressed in milliliters and the corresponding tidal impedance variation, both measured at previous PEEP level.

Furthermore, we evaluated for each patient the PEEP level providing the minimal percentage of silent spaces (Table S1).

**Lung mechanics measurements.** A heated pneumotachograph (Fleisch type 2, Fleisch, Lausanne, Switzerland) was used to measure flow. The pneumotachograph was linear over the range of flows used in this study. Volumes (VT) were obtained by time integration of the flow signal. The pressure signal was recorded at the airway opening (Pao) via a rigid polyethylene catheter connected to a differential pressure transducer (200B, Raytech instruments inc., Vancouver, B.C., Canada). Data sampling were 100 Hz. Data was recorded and analyzed offline to obtain the following:

1. Pressure-Volume curves.The P-V curve was determined by using the *constant flow method*[E15]implying a continuous low flow lung inflation. A third-degree polynomial (ΔV = a+b*Pao+c*Pao2) was fitted to the P-V curves. Factors a,b and c are constants and were determined in a non-linear least square manner using Matlab (MathWorks, Massachusetts, USA) curve fitting toolbox [E16].
2. Delta end expiratory lung volume (∆EELV) was computed as the difference between the volume in the lung at a defined PEEP level and relaxation volume on zero PEEP [E15, E16] and represents the extra-volume determined by a defined pressure of the respiratory system at functional residual capacity (FRC). ∆EELV was assessed for each PEEP level in each patient after recording of P-V curve by reducing the respiratory rate to the lowest possible value and removing PEEP as previously described [E15, E16].
3. Recruitment/derecruitment. The recruitment of previously collapsed alveoli as a result of increasing PEEP (recruited volume) or following the recruitment maneuver was identified as the upward shift along the volume axis of the P-V curve on PEEP relative to the curve on the previous PEEP level and was quantified as the increase of volume at the same Pao (20 cmH2O) [E16]. Derecruitment was identified as the downward shift of the curve during decreasing PEEP levels. Recruited/derecruited volume was expressed as the difference of volume in the lungs referring to the previous stage at the same level of Pao.Recruited volume and derecruited volumes were defined as positive or negative volume differences, respectively.
4. Respiratory system compliance (Crs), was calculated as: Crs = VT / (Pplat – PEEPtot). Pplat and PEEPtot being the airway pressure at the end of an end-inspiratory and end-expiratory hold lasting 5 seconds respectively.

**Statistical analysis.** Sample size was calculated assuming correlation coefficient between changes in lung volumes assessed by EIT and spirometry with a type I error rate of 0.01 and type II error rate of 0.20 (80% power) using the “Sampling correlation coefficient” test from Medcalc (MedCalc software, Mariakerke, Belgium). The estimated correlation coefficient used in our sample size calculation was based on a previous study (r=0.92) [E17]. Thus, a minimum of 8 patients was required. We planned to enroll an additional 30% of the estimated patient count to account for patients not able to complete the entire protocol. The total number of patients to be recruited was therefore 14 patients.

A non-normal distribution was assumed due to the low sample size (n <30). Data are presented as median and interquartile range (IQR). The Friedman repeated measures non-parametric test was used to analyze differences between the 5 phases. If a significant difference appeared, conditions were compared pairwise using the Wilcoxon test with the Bonferroni correction. Linear regression and Spearman coefficient (Rs) were calculated to evaluate potential correlations between variables.

Statistical analyses were performed using SPSS 20.0 statistical software (SPSS Inc., Chicago, IL, USA). In all statistical analyses, a two-tailed test was performed and the p-value equal or less than 0.05 was considered statistical significant.

**ADDITIONAL DATA ANALYSIS**

**Overinflation and Collapse evaluated using the relative pixel compliance technique change**

The analysis was conducted according to the paper from Costa et al. [E18] using a proper made Matlab (MATLAB R2016a, MathWorks, Inc., Natick) script. Specifically:

1. A tidal EIT image for 10 consecutive breaths was exported for each patient for each PEEP step. The 10 breaths were averaged in one representative breath.
2. For each representative breath, knowing the respiratory system driving pressure, we calculated the regional compliance at the pixel level using the following formula
3. The recruitable alveolar collapse (Collapse) and the estimation of alveolar hyperdistension (Hyperinflated areas) were obtained from the formulas (2), (3), (4) and (5) from Costa et al. considering the relative variation from each local (=regional) best compliance.
4. The analysis was conducted only on the decremental phase of our protocol, since the hypothesis behind the mathematical model by Costa et al. requires a decremental positive Positive End Expiratory Pressure trial.
5. Since, by definition, the Costa et al. is a model based on relative differences, the starting point of the trial is supposed to have 0 collapsed lung (PEEP 15 in our case) such as the last step is supposed to have 0 hyperinflation (PEEP5b in our protocol).

The average level of relative overdistention at the three different PEEP steps was respectively 24.7 ±14%, 8.2±7.5% and 0%. The average levels of reversible collapse was, instead, 0%, 4±6.3% and 13.9±8.9%. We found a big amount of heterogeneity in our population (Individual data are reported in Table S4).

In our manuscript we suspected overdistention related to higher PEEP levels by evaluating the regional compliance of the non-dependent lung. This data was confirmed by this analysis: the increase of non-dependent regional compliance with the reduction of PEEP in the decremental step of the protocol was associated with a reduction of hyperinflated areas (Figure S2).

**REFERENCES**

E1. ARDS definition Task Force et al. Acute respiratory distress syndrome: the Berlin Definition. *JAMA* 2012; 307:2526-33.

E2. Acute Respiratory Distress Syndrome Network. Ventilation with lower tidal volumes as compared with traditional tidal volumes for acute lung injury and the acute respiratory distress syndrome. *N Engl J Med* 2000; 4;342(18):1301-8.

E3. Ferrario D, Grychtol B, Adler A, et al. Toward morphological thoracic EIT: major signal sources correspond to respective organ locations in CT. *IEEE Trans Biomed Eng* 2012; 59(11):3000-8.

E4. Constantin JM, Grasso S, Chanques G, et al. Lung morphology predicts response to recruitment maneuver in patients with acute respiratory distress syndrome. *Crit Care Med* 2010; 38(4):1108-17.

E5. Borges JB, Okamoto VN, Matos GF, et al. Reversibility of lung collapse and hypoxemia in early acute respiratory distress syndrome. *Am J Respir Crit Care Med* 2006; 174(3):268-78.

E6. Crotti S, Mascheroni D, Caironi P, et al. Recruitment and derecruitment during acute respiratory failure: a clinical study. *Am J Respir Crit Care Med* 2001; 164(1):131-40.

E7. Adler A, Arnold JH, Bayford R, et al. GREIT: a unified approach to 2D linear EIT reconstruction of lung images. *Physiol Meas* 2009; 30(6):S35-55.

E8. Ukere A, März A, Wodack KH, et al. Perioperative assessment of regional ventilation during changing body positions and ventilation conditions by electrical impedance tomography. *Br J Anaesth* 2016; 117(2):228-35.

E9. Frerichs I, Hahn G, Golisch W, et al. Monitoring perioperative changes in distribution of pulmonary ventilation by functional electrical impedance tomography. *Acta Anaesthesiol Scand* 1998; 42:721-6.

E10. Radke OC, Schneider T, Heller AR, et al. Spontaneous breathing during general anesthesia prevents the ventral redistribution of ventilation as detected by electrical impedance tomography: a randomized trial. *Anesthesiology* 2012; 116:1227-34.

E11. Frerichs I, Amato MB, van Kaam AH, et al. TREND study group. Chest electrical impedance tomography examination, data analysis, terminology, clinical use and recommendations: consensus statement of the TRanslational EIT developmeNt stuDy group. *Thorax* 2017; 72(1):83-93.

E12. Zick G, Elke G, Becher T, et al. Effect of PEEP and tidal volume on ventilation distribution and end-expiratory lung volume: a prospective experimental animal and pilot clinical study. *PLoS One* 2013; 8(8):e72675.

E13. Suarez-Sipmann F, Böhm SH, Tusman G, et al. Use of dynamic compliance for open lung positive end-expiratory pressure titration in an experimental study. *Crit Care Med* 2007; 35(1):214-21.

E14. Mauri T, Eronia N, Turrini C, et al. Bedside assessment of the effects of positive end-expiratory pressure on lung inflation and recruitment by the helium dilution technique and electrical impedance tomography. *Intensive Care Med* 2016; 42(10):1576-87.

E15. Ranieri VM, Eissa NT, Corbeil C, et al. Effect of PEEP on alveolar recruitment and gas exchange in ARDS patients. *Am Rev Respir Dis* 1991; 144:538-543.

E16. Ranieri VM, Giuliani R, Fiore T, et al. Volume-pressure curve of the respiratory system predicts effects of PEEP in ARDS: "Occlusion" versus "constant flow" technique. *Am J Respir Crit Care Med* 1994; 149:19-27.

E17. Grivans C, Lundin S, Stenqvist O, et al. Positive end-expiratory pressure-induced changes in end-expiratory lung volume measured by spirometry and electric impedance tomography. *Acta Anaesthesiol Scand* 2011; 55(9):1068-77.

E18. Costa EL, Borges JB, Melo A, et al. Bedside estimation of recruitable alveolar collapse and hyperdistension by electrical impedance tomography. *Intensive Care Med* 2009; 35(6):1132-7.

**ADDITIONAL FIGURE LEGENDS**

**Figure S1**. **Study protocol.** All patients underwent five different phases, each lasting 20 minutes: positive end-expiratory pressure PEEP 5 cmH2O incremental phase; PEEP 10 cmH2O incremental phase; recruitment maneuver (RM) + PEEP 15 cmH2O; PEEP 10 cmH2O decremental phase and PEEP 5 cmH2O decremental phase. At each phase, we performed a pressure-volume curve (P-V curve) and measured delta end-expiratory lung volume (ΔEELV).

**Figure S2. Hyperinflation and non-dependent regional compliance.** The increase of non-dependent regional compliance with the reduction of PEEP in the decremental step of the protocol was associated with a reduction of hyperinflated areas estimated with the method from Costa et al [E18].

**ADDITIONAL TABLES**

**Table S1. PEEP levels corresponding to the lowest value of Total Silent Spaces (SSTOT), Dependent Silent Spaces (DSS) and Non-dependent Silent Spaces (NSS) and to the best value of TDI. * = decremental PEEP phase.**

| **Patient** |  | **SStot (%)** | **DSS (%)** | **NSS (%)** | **1-TDI** |
| --- | --- | --- | --- | --- | --- |
| **1** | *Best value* | 13 | 9 | 3 | 0,04 |
|  | *Corresponding PEEP* | 10* | 10 | 10* | 10* |
| **2** | *Best value* | 18 | 13 | 2 | 0,10 |
|  | *Corresponding PEEP* | 15 | 15 | 5* | 15 |
| **3** | *Best value* | 9 | 4 | 0 | 0,07 |
|  | *Corresponding PEEP* | 10* | 15 | 5 | 5 |
| **4** | *Best value* | 9 | 7 | 0 | 0,06 |
|  | *Corresponding PEEP* | 15 | 15 | 5 | 15 |
| **5** | *Best value* | 13 | 11 | 2 | 0,39 |
|  | *Corresponding PEEP* | 15 | 15 | 10* | 15 |
| **6** | *Best value* | 12 | 9 | 3 | 0,12 |
|  | *Corresponding PEEP* | 5* | 10* | 5* | 15 |
| **7** | *Best value* | 9 | 8 | 1 | 0,07 |
|  | *Corresponding PEEP* | 15 | 15 | 5* | 10 |
| **8** | *Best value* | 14 | 13 | 1 | 0,31 |
|  | *Corresponding PEEP* | 15 | 15 | 5 | 15 |
| **9** | *Best value* | 7 | 5 | 0 | 0,03 |
|  | *Corresponding PEEP* | 15 | 15 | 5/10 | 15 |
| **10** | *Best value* | 3 | 3 | 0 | 0,01 |
|  | *Corresponding PEEP* | 15 | 15 | 10/15/10* | 10 |
| **11** | *Best value* | 7 | 7 | 0 | 0,14 |
|  | *Corresponding PEEP* | 15 | 15 | All | 15 |
| **12** | *Best value* | 7 | 6 | 0 | 0,47 |
|  | *Corresponding PEEP* | 15 | 15 | 5/10 | 15 |
| **13** | *Best value* | 16 | 16 | 0 | 0,27 |
|  | *Corresponding PEEP* | 15 | 15 | 5* | 10 |
| **14** | *Best value* | 19 | 11 | 0 | 0,31 |
|  | *Corresponding PEEP* | 15 | 15 | 5* | 15 |

|  | **Driving Pressure (cmH2O)** | | | | | **Compliance of the respiratory system (ml/cmH2O)** | | | | |
| --- | --- | --- | --- | --- | --- | --- | --- | --- | --- | --- |
| ID | *PEEP5 incremental phase* | *PEEP10 incremental phase* | *RM + PEEP 15* | *PEEP10 decremental phase* | *PEEP5 decremental phase* | *PEEP5 incremental phase* | *PEEP10 incremental phase* | *RM + PEEP 15* | *PEEP10 decremental phase* | *PEEP5 decremental phase* |
| 1 | 11.4 | 13.7 | 12.7 | 12.8 | 14.7 | 39.4 | 32.8 | 35.4 | 35.3 | 30.6 |
| 2 | 14.0 | 12.8 | 12.8 | 11.5 | 11.6 | 39.3 | 42.8 | 42.9 | 47.9 | 47.3 |
| 3 | 10.3 | 9.8 | 8.5 | 8.0 | 8.2 | 43.5 | 46.0 | 53.1 | 56.4 | 54.6 |
| 4 | 10.6 | 10.6 | 11.8 | 11.1 | 11.0 | 37.7 | 37.8 | 34.0 | 36.2 | 36.4 |
| 5 | 9.8 | 8.4 | 8.5 | 7.2 | 7.9 | 50.8 | 59.3 | 58.9 | 69.1 | 63.4 |
| 6 | 8.9 | 9.6 | 11.0 | 8.4 | 8.1 | 54.9 | 51.1 | 44.6 | 58.6 | 60.4 |
| 7 | 10.5 | 10.5 | 11.8 | 9.3 | 8.5 | 38.0 | 38.1 | 33.9 | 42.9 | 46.9 |
| 8 | 10.1 | 11.6 | 12.4 | 9.3 | 9.3 | 40.5 | 35.2 | 33.1 | 44.1 | 44.3 |
| 9 | 16.8 | 18.2 | 18.5 | 16.1 | 15.4 | 23.9 | 21.9 | 21.6 | 24.9 | 25.9 |
| 10 | 15.0 | 10.3 | 10.4 | 10.7 | 12.9 | 26.0 | 37.8 | 37.5 | 36.6 | 30.4 |
| 11 | 9.1 | 11.2 | 11.8 | 9.6 | 9.7 | 49.3 | 40.2 | 38.2 | 46.9 | 46.4 |
| 12 | 15.4 | 11.6 | 11.2 | 12.2 | 14.8 | 24.7 | 32.7 | 33.9 | 31.2 | 25.7 |
| 13 | 14.5 | 14.6 | 11.7 | 14.7 | 16.1 | 34.4 | 34.2 | 42.8 | 34.1 | 31.0 |
| 14 | 9.6 | 10.4 | 10.6 | 7.5 | 8.5 | 45.9 | 42.5 | 41.7 | 58.8 | 51.6 |

**Table S2: individual values of respiratory system driving pressure (cmH2O) and respiratory system compliance (ml/cmH2O) during the trial.**

**Table S3: individual values of Dependent and Non-dependent Silent spaces (% of total pixels) during the trial.**

|  | **Dependent Silent Spaces (% of total pixels)** | | | | | **Non-dependent Silent Spaces (% of total pixels)** | | | | |
| --- | --- | --- | --- | --- | --- | --- | --- | --- | --- | --- |
| ID | *PEEP5 incremental phase* | *PEEP10 incremental phase* | *RM + PEEP 15* | *PEEP10 decremental phase* | *PEEP5 decremental phase* | *PEEP5 incremental phase* | *PEEP10 incremental phase* | *RM + PEEP 15* | *PEEP10 decremental phase* | *PEEP5 decremental phase* |
| 1 | 10.90 | 9.43 | 9.44 | 10.43 | 12.62 | 12.62 | 5.93 | 5.77 | 2.92 | 11.90 |
| 2 | 16.31 | 14.11 | 13.09 | 15.68 | 17.00 | 4.15 | 3.88 | 4.41 | 3.22 | 2.02 |
| 3 | 8.82 | 5.96 | 4.41 | 5.90 | 6.83 | 0.08 | 3.27 | 4.29 | 2.62 | 3.16 |
| 4 | 14.56 | 9.63 | 7.36 | 8.65 | 12.33 | 0.01 | 0.29 | 1.31 | 0.91 | 0.73 |
| 5 | 20.08 | 17.19 | 10.52 | 12.34 | 14.96 | 5.47 | 5.48 | 2.35 | 1.77 | 2.07 |
| 6 | 26.10 | 14.99 | 9.49 | 8.82 | 9.40 | 3.06 | 4.07 | 4.89 | 3.68 | 2.60 |
| 7 | 16.99 | 10.13 | 7.98 | 10.38 | 16.00 | 0.70 | 1.31 | 1.41 | 1.58 | 0.73 |
| 8 | 15.13 | 15.99 | 12.69 | 13.89 | 17.70 | 1.08 | 1.20 | 1.79 | 1.97 | 1.84 |
| 9 | 9.30 | 8.37 | 5.24 | 5.74 | 6.91 | 0.00 | 0.00 | 1.27 | 1.24 | 0.42 |
| 10 | 12.00 | 5.50 | 3.47 | 5.20 | 8.41 | 0.15 | 0.00 | 0.00 | 0.00 | 0.17 |
| 11 | 17.22 | 11.63 | 6.87 | 9.35 | 13.52 | 0.00 | 0.00 | 0.00 | 0.00 | 0.00 |
| 12 | 16.98 | 12.90 | 6.49 | 11.51 | 14.10 | 0.00 | 0.00 | 0.39 | 0.00 | 0.02 |
| 13 | 40.78 | 29.28 | 15.91 | 30.62 | 35.62 | 0.08 | 3.08 | 0.41 | 3.06 | 0.04 |
| 14 | 16.38 | 13.36 | 10.58 | 12.05 | 13.75 | 0.70 | 0.43 | 1.47 | 0.63 | 0.20 |

**Table S4: Individual data and mean (±SD) of lung collapse and overdistension for each step of the protocol evaluated trough the relative pixel compliance variation technique.**

| ID | **Hyperinflation 15 (%)** | **Hyperinflation 10b (%)** | **Hyperinflation 5b (%)** | **Collapse 15 (%)** | **Collapse 10b (%)** | **Collapse 5b (%)** |
| --- | --- | --- | --- | --- | --- | --- |
| 1 | 21.2 | 22.1 | 0.0 | 0.0 | 1.3 | 18.0 |
| 2 | 26.4 | 5.4 | 0.0 | 0.0 | 1.8 | 8.3 |
| 3 | 20.7 | 4.5 | 0.0 | 0.0 | 3.2 | 11.5 |
| 4 | 30.9 | 14.3 | 0.0 | 0.0 | 1.1 | 7.9 |
| 5 | 22.9 | 3.4 | 0.0 | 0.0 | 0.6 | 5.8 |
| 6 | 42.9 | 20.5 | 0.0 | 0.0 | 0.9 | 8.2 |
| 7 | 41.5 | 14.6 | 0.0 | 0.0 | 0.1 | 11.2 |
| 8 | 30.4 | 3.2 | 0.0 | 0.0 | 0.4 | 7.5 |
| 9 | 21.1 | 8.6 | 0.0 | 0.0 | 1.3 | 11.6 |
| 10 | 4.3 | 0.1 | 0.0 | 0.0 | 7.4 | 30.9 |
| 11 | 34.6 | 13.7 | 0.0 | 0.0 | 2.1 | 8.9 |
| 12 | 1.9 | 0.4 | 0.0 | 0.0 | 16.3 | 32.4 |
| 13 | 4 | 1.12 | 0.0 | 0.0 | 20.2 | 24.12 |
| 14 | 43.6 | 2.4 | 0.0 | 0.0 | 0.1 | 8.0 |
|  | | | | | | |
| *Mean* | *24.7* | *8.2* | *0.0* | *0.0* | *4.0* | *13.9* |
| *SD* | *14.0* | *7.5* | *0.0* | *0.0* | *6.3* | *8.9* |
