# Supplementary figures and images for: Variation of poorly ventilated lung units (silent spaces) measured by electrical impedance tomography to dynamically assess recruitment
Source: Crit Care. 2018 Jan 31;22:26. doi: 10.1186/s13054-017-1931-7 (PMC5793388; doi:10.1186/s13054-017-1931-7)

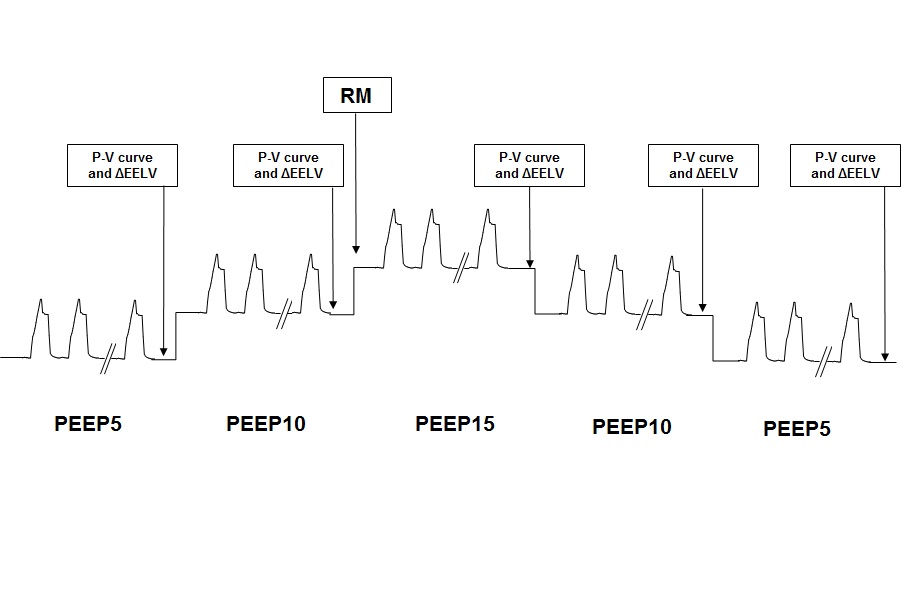

Supplement: Additional file 1: — Variation of poorly ventilated lung units (silent spaces) measured by electrical impedance tomography to dynamically assess recruitment. Additional information about the manuscript methods and additional data analysis are provided. Figure S1. Study protocol. Study protocol consisted of five consecutive phases. Figure S2. Hyperinflation (%) and nondependent lung compliance (ml/cmH2O) during the decremental step of the protocol. Hyperinflation (%) and nondependent lung compliance (ml/cmH2O) during the decremental step of the protocol. The hyperinflation value is expressed as a percentage of the total pixels and is relative to the last step of the PEEP titration trial (in this case, PEEP = 5 cmH2O). (ZIP 178 kb) [file 13054_2017_1931_MOESM1_ESM.zip › Figure S1.jpg]

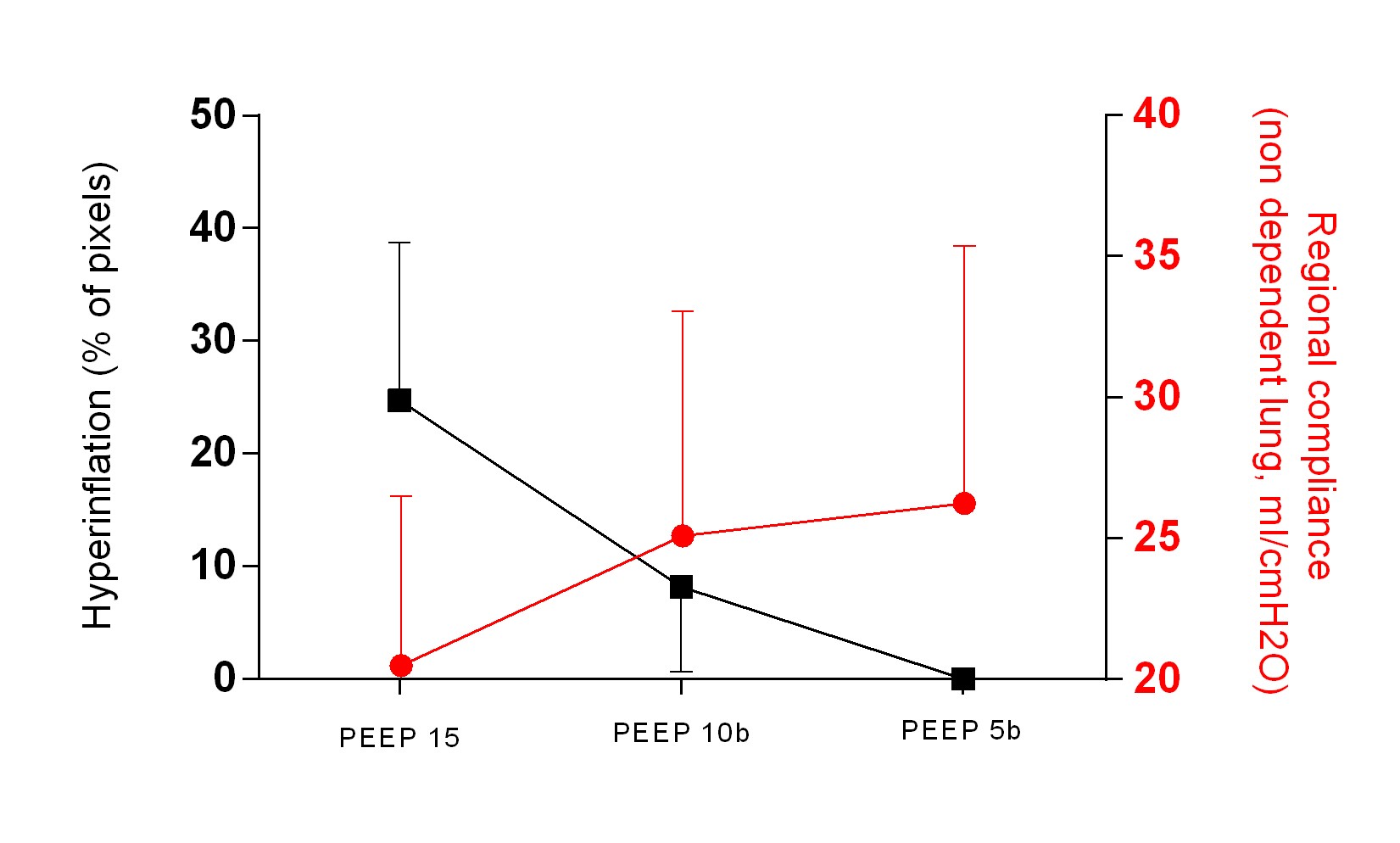

Supplement: Additional file 1: — Variation of poorly ventilated lung units (silent spaces) measured by electrical impedance tomography to dynamically assess recruitment. Additional information about the manuscript methods and additional data analysis are provided. Figure S1. Study protocol. Study protocol consisted of five consecutive phases. Figure S2. Hyperinflation (%) and nondependent lung compliance (ml/cmH2O) during the decremental step of the protocol. Hyperinflation (%) and nondependent lung compliance (ml/cmH2O) during the decremental step of the protocol. The hyperinflation value is expressed as a percentage of the total pixels and is relative to the last step of the PEEP titration trial (in this case, PEEP = 5 cmH2O). (ZIP 178 kb) [file 13054_2017_1931_MOESM1_ESM.zip › Figure S2.jpg]
